# The Gut Microbiome and Abiotic Factors as Potential Determinants of Postprandial Glucose Responses: A Single-Arm Meal Study

**DOI:** 10.3389/fnut.2020.594850

**Published:** 2021-01-14

**Authors:** Nathalie Nestel, Josephine D. Hvass, Martin I. Bahl, Lars H. Hansen, Lukasz Krych, Dennis S. Nielsen, Lars Ove Dragsted, Henrik M. Roager

**Affiliations:** ^1^Department of Nutrition, Exercise and Sports, University of Copenhagen, Frederiksberg, Denmark; ^2^National Food Institute, Technical University of Denmark, Kgs. Lyngby, Denmark; ^3^Department of Plant and Environmental Science, University of Copenhagen, Frederiksberg, Denmark; ^4^Department of Food Science, University of Copenhagen, Frederiksberg, Denmark

**Keywords:** gut microbiome, personalized nutrition, individuality, intestinal transit time, abiotic factors, colonic fermentation

## Abstract

The gut microbiome has combined with other person-specific information, such as blood parameters, dietary habits, anthropometrics, and physical activity been found to predict personalized postprandial glucose responses (PPGRs) to various foods. Yet, the contributions of specific microbiome taxa, measures of fermentation, and abiotic factors in the colon to glycemic control remain elusive. We tested whether PPGRs 60 min after a standardized breakfast was associated with gut microbial α-diversity (primary outcome) and explored whether postprandial responses of glucose and insulin were associated with specific microbiome taxa, colonic fermentation as reflected by fecal short-chain fatty acids (SCFAs), and breath hydrogen and methane exhalation, as well as abiotic factors including fecal pH, fecal water content, fecal energy density, intestinal transit time (ITT), and stool consistency. A single-arm meal trial was conducted. A total of 31 healthy (24 female and seven male) subjects consumed a standardized evening meal and a subsequent standardized breakfast (1,499 kJ) where blood was collected for analysis of postprandial glucose and insulin responses. PPGRs to the same breakfast varied across the healthy subjects. The largest inter-individual variability in PPGRs was observed 60 min after the meal but was not associated with gut microbial α-diversity. In addition, no significant associations were observed between postprandial responses and specific taxa of the gut microbiome, measures of colonic fermentation, ITT, or other abiotic factors. However, fasting glucose concentrations were negatively associated with ITT, and fasting insulin was positively associated with fasting breath hydrogen. In conclusion, the gut microbiome, measures of colonic fermentation, and abiotic factors were not shown to be significantly associated with variability in postprandial responses, suggesting that contributions of the gut microbiome, colonic fermentation, and abiotic factors to PPGRs may be subtle in healthy adults.

## Introduction

Elevated postprandial glucose response (PPGR) is associated with type 2 diabetes mellitus (T2DM), which is a worldwide growing concern (1). Previous research has uncovered high variability in postprandial glucose and insulin responses of different people to the same food (1–5), supporting the need for personalized nutrition in contrast to the prevalent “one size fits all” approach to dietary guidance. Recent studies (3–5) have found that the gut microbiome composition is associated with variations in PPGR to different foods. However, the contribution of specific bacterial taxa of the gut microbiome to the variability in PPGR and the underlying mechanisms remain elusive. High gut microbial α-diversity has been suggested as a marker of a healthy gut (6, 7), which is associated with intake of a high diversity of vegetables and fruits (8). However, high gut microbial α-diversity can be confounded by a long intestinal transit time (ITT) (9, 10), which favors growth of slow-growing species and thereby increase microbial richness (11). Abiotic factors such as pH, ITT, and stool consistency are determinants of gut microbial composition, diversity, and metabolism (9–12). Furthermore, ITT is a determinant of stool pH and short-chain fatty acid (SCFA) concentrations (13), as well as microbial hydrogen and methane production in the colon (14). Together, this emphasizes that abiotic factors are fundamental determinants of the gut microbial composition and metabolism, suggesting that individual differences in abiotic factors could explain differences in the gut microbiome and be associated with variations in PPGR. Therefore, we hypothesized as the primary outcome that the individual PPGR 60 min after a standardized meal test is inversely associated with gut microbial diversity in healthy subjects. In addition, we explored whether PPGR after a standardized meal is associated with baseline gut microbial composition and metabolism as reflected by fecal SCFAs and breath hydrogen and methane exhalation, as well as abiotic factors including fecal pH, fecal water content, fecal energy density, ITT, and stool consistency assessed by the Bristol Stool Scale (BSS).

## Materials and Methods

### Protocol

The study (MIGLUCOSE) was conducted as a single-arm meal study at the Department of Nutrition, Exercise and Sports, University of Copenhagen, Denmark, from October to December 2018. The study was approved by The Ethical Committee of the Capital Region of Denmark (H-18032846) and conducted according to the Declaration of Helsinki, and the handling of personal data was endorsed by the Faculty of Science, University of Copenhagen (514-0052/18-5000). All subjects signed an informed consent form before participating in the study. The study was registered at ClinicalTrials.gov (ID: NCT03686293).

### Participants

A total of 31 healthy Danish subjects (24 women and seven men) were recruited and completed the test day. The inclusion criteria were as follows: male and female; aged 18–40 years; body mass index (BMI) <27 kg/m^2^; no history of chronic or infectious diseases; no medical conditions and not taking medication known to influence any of the outcome measures; no blood donations or participation in another scientific study within the three previous months; no oral antibiotics, diarrhea inhibitors, or laxatives taken within the previous six months; and no pregnant or lactating women. The inclusion criteria regarding age and BMI were chosen to target a rather homogenous and healthy group of adults. The participants were recruited from October until December 2018 through poster boards at educational institutions in Copenhagen and on the websites www.forsøgsperson.dk, https://nexs.ku.dk/om_nexs/forsogspersoner/, and www.sundhed.dk and via social media (Twitter, Facebook, Instagram, and LinkedIn). One participant turned 41 years between the period of giving written informed consent and the study day.

### Prior to the Meal Test

Ten days prior to the test day, subjects were asked not to consume any sweet corn (maize). Five days prior to the test day, subjects were asked to consume 100 g of provided sweet corn with no other foods, 2 h before dinner. In the subsequent five consecutive days, the subjects filled out a defecation diary where they noted down the time and date of the sweet corn consumption and the time and date of when they observed sweet corn excreted in their feces. Based on this, the subjects' ITT was estimated.

The subjects were asked to abstain from alcohol intake, strenuous exercise, and painkillers containing paracetamol 24 h before the test day. The participants were asked to consume a standardized meal on the evening before the test day, which they prepared according to a provided recipe (wheat spaghetti with lentils and tomato sauce), followed by at least 12 h of fasting where they were only allowed to consume 500 ml of water. Finally, participants were instructed to collect a fecal sample between 4 p.m. the day before the test day and 8 a.m. on the morning of the test day. However, one participant handed in a fecal sample collected in the morning the day before the test day, and one subject delivered the fecal sample the morning after the test day. Subjects assessed the fecal consistency by the BSS, which is a validated surrogate measure for gastrointestinal transit time (15).

### Meal Test

On the test day, the participants were asked to refrain from brushing their teeth and smoking. Avoidance of smoking was to avoid the contamination of exogenous hydrogen from cigarette smoke when conducting the breath exhalation measurements. The avoidance of teeth brushing was done to preserve saliva samples, which were collected for microbiome profiling but not included in the present study. Additionally, the participants were instructed to arrive at the study site using the least strenuous form of transportation. On arrival at the study site, subjects delivered the collected fecal sample, which had been kept cold in a cooling bag with freeze elements upon collection at home and during transportation to the laboratory. In the fasting condition, anthropometric measurements (height and body weight) were obtained, and a peripheral venous catheter was placed inside the elbow joint of the subjects from which blood could be drawn. Each participant consumed 500 mg of paracetamol (1 × 500 mg tablet, Panodil, GlaxoSmithKline Dungarvan Ltd.), allowing for measurement of gastric emptying (16) and 150 ml water followed by a carbohydrate rich standardized breakfast (1,499 kJ, Supplementary Table 1) consisting of two slices of white toast bread (60 g) with butter (8 g) and jam (20 g) and 250 ml of orange juice (macronutrient breakdown: 60.9 g of carbohydrate, 9.1 g of fat, and 6.6 g of protein). The participants were asked to consume the test meal within 15 min. Blood samples were drawn into EDTA plasma (for glucose determination) and serum (for insulin determination and mass spectrometry) tubes before the meal (fasting) and at 15, 30, 60, 90, and 120 min after starting the test meal. Finally, breath exhalation samples were collected before the meal (fasting) by exhaling air into a provided sampling bag.

### Compliance

Compliance to the standardization procedures was evaluated by self-assessment questionnaires on the test day. In addition, to assess whether the participants had in fact consumed the standardized meal including lentils the evening before the test day, we performed ultra-performance liquid chromatography–mass spectrometry (UPLC-MS) on serum samples as previously published (17) and checked for the presence of tryptophan betaine (247.1448 [M + H], retention time 2.85 min), a biomarker of chickpeas and lentils peaking in blood 4–6 h after consumption before slowly being excreted (18). Tryptophan betaine was detected in considerable amounts in all serum samples except from one male subject (Supplementary Figure 1), suggesting that all participants except one had been compliant and consumed the standardized evening meal containing lentils. The non-compliant subject was excluded from further analyses.

### Biochemical Analysis of Blood

EDTA plasma samples were upon collection immediately put on ice, until they were centrifuged for precipitation of blood cells and stored at −80°C. The serum samples were left to clot at room temperature for 30 min, and the supernatants were collected into cryotubes and stored at −80°C. Glucose was measured in plasma samples by using Pentra ABX 400 (HORIBA ABX, Montpellier, France). The detection limit was 0.11 mmol/L, and the reference interval for fasting glucose was 4.2–6.3 mmol/L. Serum insulin levels were measured by using Immulite 2000 XPi (Siemens Healthcare Diagnostics Ltd., Llaneris Gwynedd LL554EL, United Kingdom). The detection limit was 14.4 pmol/L. Measurements below detection limit were set to half the detection limit (7.2 pmol/L). Prior to analysis of insulin and glucose, both the instrument's performances were validated using external and internal insulin and glucose controls. For the external glucose controls, the coefficient of variation (CV) of glucose was 1.2% [low-level control (5.3–5.5 mmol/L)] and 0.7% [high-level control (14.5–14.8 mmol/L)]. For the internal glucose controls (range 4.5–4.7 mmol/L), the CV was 1.2%. For the external insulin controls, the CV of insulin was 2.0% [low-level control (70.1–74.3 pmol/L)] and 3.3% [high-level control (358.0–397.0 pmol/L)]. For the internal insulin controls (range 48.9–56.7 pmol/L), the CV was 4.1%.

### Breath Exhalation Measurements

Concentrations of hydrogen and methane were measured in all breath samples using the M.E.C. Lactotest 202 Xtend (M.E.C. R&D sprl, Brussels, Belgium), as a proxy of colonic fermentation (19).

### Fecal Measurements

Fecal samples were homogenized in sterile water 1:1, and pH was determined using a digital pH meter (Lutron PH-208, Taiwan). The homogenized samples were aliquoted to cryotubes and stored at −80°C. SCFAs were quantified in feces by UPLC-MS as previously described (17). Fecal water content was determined by drying fecal samples for 48 h at 50°C or until complete dryness. Gross energy density of fecal samples was determined by combusting ~150 mg of dry feces in a bomb calorimeter C6000 (IKA, Staufen, Germany) using benzoic acid as a calibrator (IKA® C 723). The energy density in the dried feces was converted into energy density per gram wet feces.

### Microbiome Profiling

DNA was extracted from ~100 mg of homogenized feces by Bead-Beat Micro AX Gravity method (A&A Biotechnology, Gdynia, Poland) with several adjustments mentioned below. A NanoDrop ND-1000 Spectrophotometer (NanoDrop Technologies Inc., Wilmington, USA) was used to assess the purity of the extracted DNA. The DNA concentration was measured using Qubit dsDNA BR Assay Kit (Thermo Fisher Scientific Inc., Waltham, USA). The gut microbiota composition of 27 subjects was successfully analyzed by 16S ribosomal RNA (rRNA) gene amplicon sequencing of the V3 region on the Illumina NextSeq platform (Illumina Inc.) with the Mid Output Kit v2 (300 cycles) as previously described (20). The raw dataset containing pair-ended reads with matching quality scores were fused and clipped in the USEARCH pipeline using fastq_mergepairs and fastq_filter scripts. The UNOISE was used to purge the dataset from chimeric reads and to build zero radius Operational Taxonomic Units (zOTUs). As a reference database, the Greengenes (13.8) 16S rRNA gene collection was employed. The acquired 16S rRNA gene amplicon data were pre-processed using the web-based tool MicrobiomeAnalyst (21). The average count per sample was 65,142 reads (ranging from 16,647 to 151,790). After zOTUs that either were low abundant (<10 counts) or had low prevalence (<10%) across samples were filtered out, 1,215 zOTUs remained. The data were then rarefied to the minimum library size (16,647 reads) to make sure that all samples were comparable. The subjects' fecal α-diversity was assessed by both the Shannon index and zOTU richness. For correlation analyses, core zOTUs (17/480) and bacterial genera (19) present in at least 30% of the samples were used.

### Statistical Analysis

Based on a pilot experiment, we expected to obtain a correlation coefficient of 0.5 between gut microbial diversity and postprandial glucose levels at 60 min. With alpha set to 0.05 and beta set to 0.20, we calculated that 29 participants would be needed. Statistical analyses were conducted in R (version 3.4.2) and GraphPad Prism (version 8.2.0). The area under the curves (AUCs) for glucose and insulin concentrations during the test period were calculated by using the trapezoidal method, where the total AUCs were determined using the entire area above zero, using the following formula: *AUC* = *x*t ((*y*1 + *y*2)/2). The *ggplot2* package (version 3.4.4) was used to calculate the 95% confidence interval (CI) for the insulin and glucose concentrations from 0 to 120 min. Correlations between baseline and postprandial glucose and insulin measures (30, 60, 90, and 120 min), individual microbiome features, and other colonic factors, such as fecal SCFAs, breath hydrogen and methane exhalation, ITT, stool consistency, fecal pH, fecal water content, and fecal energy density, were calculated using the standard Spearman's rank correlation, as implemented in the *ppcor* R package (version 3.4.4) (22). Random forest predictions of fasting and postprandial glucose and insulin responses by microbiota and abiotic variables composed of 1,001 trees and were computed using the default settings of the randomForest function implemented in the randomForest R package (23). For explorative correlation analyses, *p*-values were adjusted for multiple testing by the Benjamini–Hochberg false discovery rate (24). A *p* < 0.05 was considered statistically significant. Heatmaps were generated in GraphPad Prism. The flow of the participants from enrolment until analysis and missing data is shown in Supplementary Figure 2.

## Results

All 31 subjects (Table 1) completed the procedures prior to the test day and ingested the test meal without any problems. However, one male subject was excluded from analysis, as tryptophan betaine, a biomarker of lentils (Supplementary Figure 1), was not detected in his blood, suggesting that this subject had not consumed the standardized evening meal prior to the test day. The 30 remaining subjects were apparently healthy. We did however notice that seven subjects had a fasting glucose in the prediabetic range (5.6–6.9 mmol/L).

**Table 1:** Clinical characteristics of the 31 healthy subjects.

**Characteristic**	**Total group^**a**^**
Sex (f/m)	24/7
Age (years)	28.4 (±5.6)
Weight (kg)	64.6 (±10.6)
Body mass index (kg/m^2^)	22.0 (±2.2)
Fasting plasma glucose (mmol/L)	5.4 (±0.3)
Fasting serum insulin (pmol/L)	35.1 (±23.9)
Intestinal transit time^b^ (h)	28.4 (±15.3)
Stool consistency^c^	3.4 (±1.3)
Fasting breath hydrogen (ppm)	31.1 (±27.6)
Fasting breath methane (ppm)	7.3 (±20.1)

a Values are means (±SD). Missing values are reported in Supplementary Figure 2.

b Intestinal transit time was estimated by the time it took sweet corn to travel through the subjects' gastrointestinal system.

c *Stool consistency was assessed by the Bristol Stool Scale, which ranges from 1 to 7, with 1 indicating hard stools and 7 indicating watery diarrhea*.

### Gut Microbiome and Postprandial Glucose and Insulin Responses

**Figure 1:**
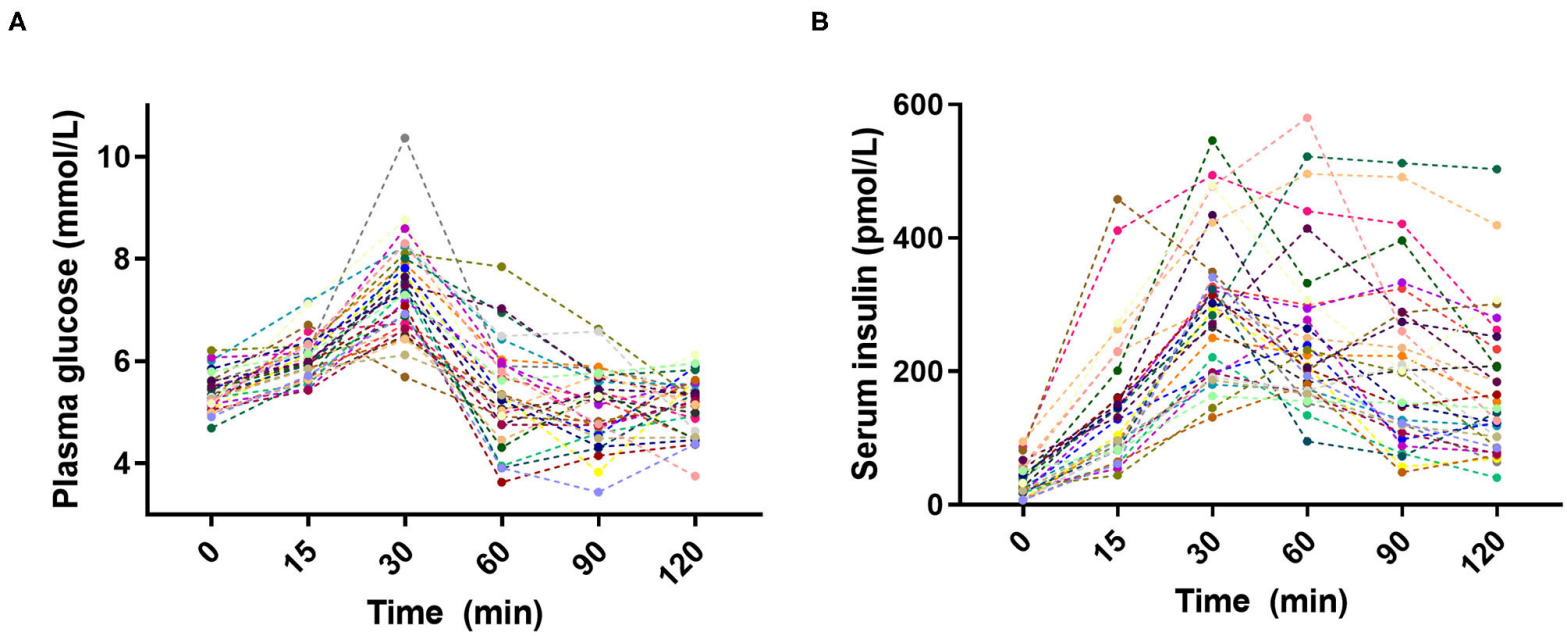
Inter-individual variation in **(A)** plasma glucose and **(B)** serum insulin postprandial responses to the standardized breakfast (*n* = 30).

Large inter-individual variations in glucose and insulin responses to the standardized breakfast were as expected observed in the 30 subjects (Figure 1). The individual glucose and insulin responses were weakly correlated across all individuals (Supplementary Figure 3), emphasizing the personal nature of glucose regulation. The inter-individual variability [as measured by the population CV (SD/mean, %)] in PPGRs was evident at all time points: 15 min (7%), 30 min (13%), 60 min (18%), 90 min (14%), and 120 min (11%). The largest inter-individual variability in PPGRs was, as hypothesized prior to the study, observed 60 min after the meal (18%), which is why we focused on this time point for the subsequent correlation analyses.

**Figure 2:**
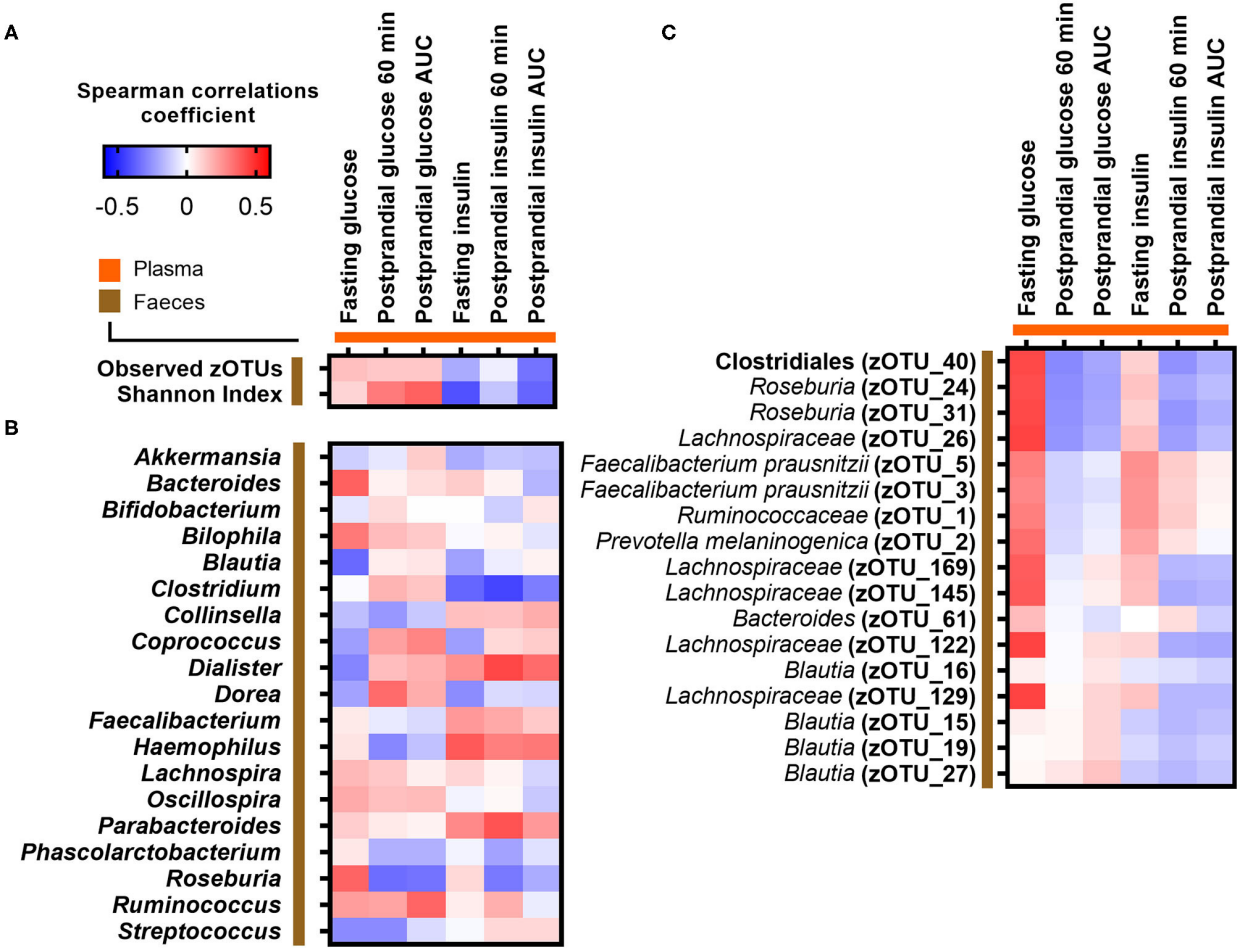
Correlations between fasting and postprandial glucose and insulin measures and **(A)** gut microbial α-diversity, **(B)** abundance of core bacterial genera, and **(C)** core zOTUs. Correlations were calculated based on Spearman's rank correlation (*n* = 27). No associations remained significant after adjustment for multiple testing.

We hypothesized prior to the study that an inverse association would exist between postprandial plasma glucose at 60 min and gut microbial α-diversity as reflected by zOTU richness. However, no correlations were observed between the PPGR after 60 min and gut microbial α-diversity as reflected by observed zOTU richness (*p* = 0.52, rho = 0.13) and Shannon index (*p* = 0.12, rho = 0.31) (Figure 2A; Supplementary Figure 4). We next investigated whether relative abundances of core bacterial genera or zOTUs (prevalent in at least 30% of subjects) were associated with fasting and postprandial glucose and insulin responses. However, no core bacterial genera or zOTUs were significantly associated with fasting or postprandial glucose and insulin responses after adjusting for multiple testing (adjusted *p* > 0.05) (Figures 2B,C).

### Associations Between Abiotic Factors, Measures of Colonic Fermentation, and Fasting and Postprandial Responses

**Figure 3:**
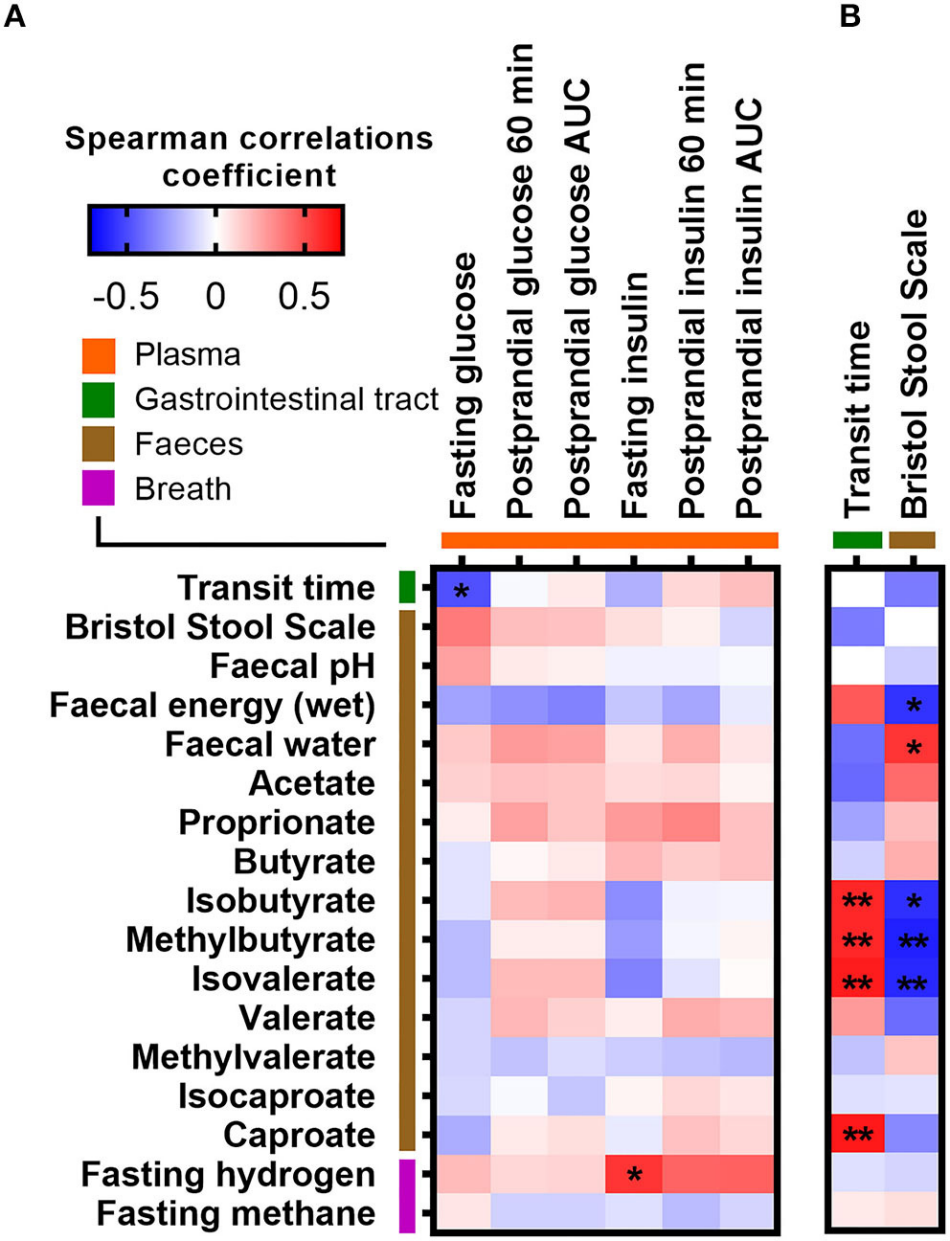
Correlations between measures of colonic fermentation, abiotic factors, and **(A**) fasting and postprandial glucose and insulin measures, as well as with **(B**) measurements of intestinal transit time, respectively. Correlations were calculated based on Spearman's rank correlation (*n* = 27). Significant associations are represented by asterisks (*adjusted *p* < 0.05, **adjusted *p* < 0.01).

We furthermore explored whether abiotic factors known to influence gut microbial composition, diversity, and metabolism were related to the fasting or postprandial state. ITT, determined by the time it took sweet corn to travel through the subjects' gastrointestinal system (median 21.5 h; range 10–55 h), was as expected negatively associated with stool consistency assessed by BSS (Spearman *r* = −0.37, *p* = 0.049; Supplementary Figure 5A) and fecal water content (Spearman *r* = −0.40, *p* = 0.029; Supplementary Figure 5B). In addition, fecal water content was positively associated with BSS (Spearman *r* = 0.56, *p* = 0.0017; Supplementary Figure 5C). Both ITT and stool consistency were consistently associated with fecal branched SCFAs (isobutyrate, methylbutyrate, and isovalerate) and fecal energy density (Figure 3). However, none of the abiotic factors (ITT, BSS, fecal water content, fecal pH, and fecal energy density) and measures of colonic fermentation (fecal SCFAs, breath hydrogen, and methane concentrations) were associated with postprandial glucose and insulin responses, respectively (Figure 3). Only ITT was negatively associated with fasting glucose (Spearman *r* = −0.49, adjusted *p* = 0.04), whereas the positive association between stool consistency and fasting glucose did not remain significant after adjustment for multiple testing (unadjusted *p* = 0.049, adjusted *p* = 0.23) (Figure 3). In addition, a positive association between fasting breath hydrogen concentrations and fasting insulin was observed (adjusted *p* = 0.016) (Figure 3). Finally, we used random forest to rank the importance of the gut microbiota and abiotic variables in predicting fasting and postprandial glucose and insulin responses. Overall, the random forest corroborated the observed correlations (Supplementary Figure 6).

### Associations Between Bacterial Genera, Short-Chain Fatty Acids, and Intestinal Transit Time

**Figure 4:**
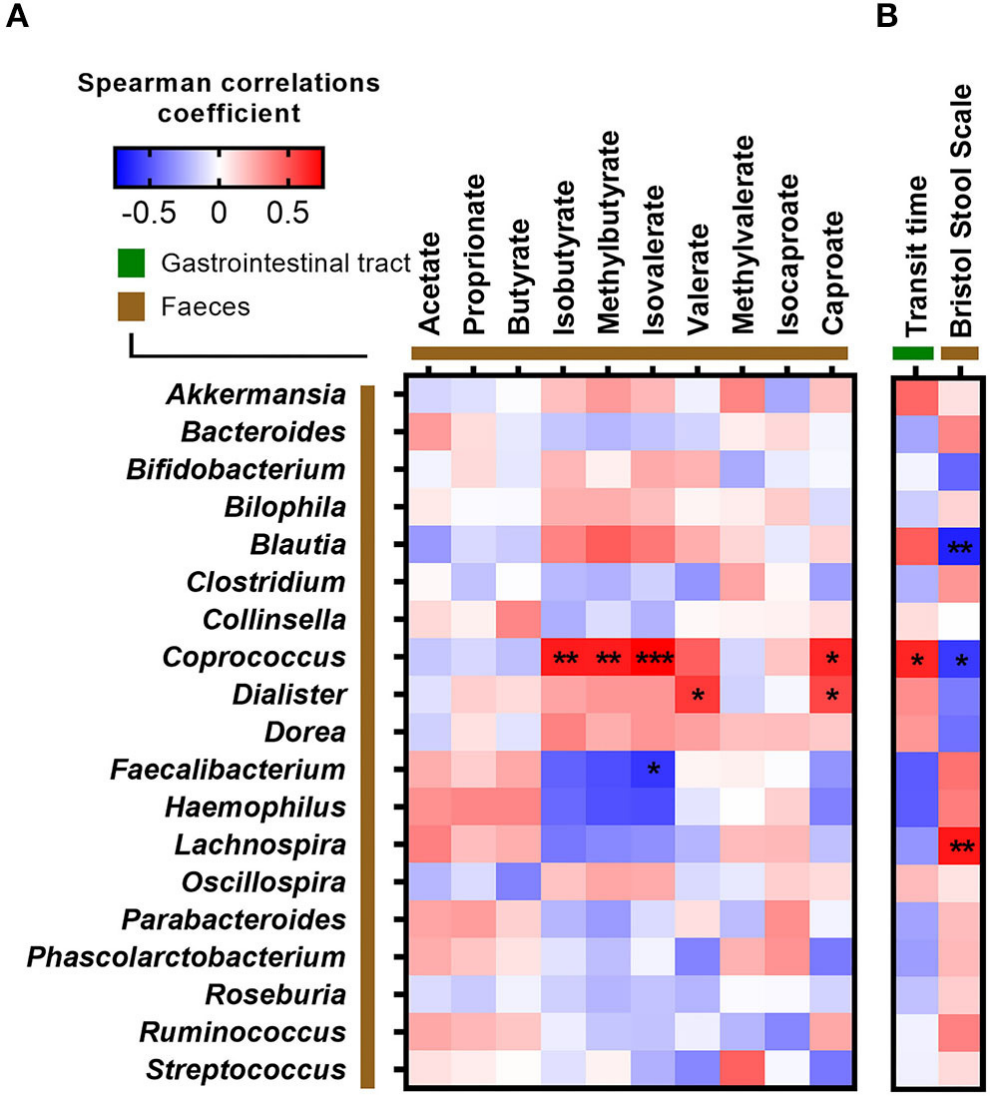
Correlations between fecal bacteria at genus level and **(A**) short-chain fatty acids and **(B**) measurements of intestinal transit time. Correlations were calculated based on Spearman's rank correlation (*n* = 27). Significant associations are represented by asterisks (*adjusted *p* < 0.05, **adjusted *p* < 0.01, ***adjusted *p* < 0.001).

Since ITT was found to associate with both fasting glucose and several branched SCFAs, we correlated ITT, stool consistency, and fecal SCFA concentrations against the relative abundance of bacterial genera (Figure 4). A longer ITT and a firmer stool were associated with higher abundance of *Coprococcus* and *Blautia* but lower abundance of *Lachnospira*. Additionally, *Coprococcus* was positively associated with the branched SCFAs isobutyrate, methylbutyrate, isovalerate, and caproate (Figures 3, 4). In contrast, the relative abundance of *Faecalibacterium* was negatively correlated with fecal isovalerate concentrations. Finally, the abundance of *Dialister* was positively associated with fecal valerate and caproate.

## Discussion

We set out to explore whether PPGRs were associated with specific taxa of the gut microbiome, measures of colonic fermentation, and abiotic factors in healthy adults. In agreement with other studies (3–5), we did observe that PPGRs are highly variable across individuals despite the recruitment of a rather homogenous group of young, healthy adults and our standardization procedures prior to the meal test. We hypothesized that the PPGR 60 min after the standardized breakfast would be inversely associated with the subjects' gut microbial diversity, which has been suggested as marker of a healthy gut (6, 7). However, we did not observe any associations between PPGRs and the gut microbial diversity nor with any other specific taxa of the gut microbiome or abiotic factors in healthy adults. This might be due to the rather small sample size of 30 individuals of which runs the risk of false negative, type 2 error. Furthermore, the fact that only six participants were males might decrease the generalizability. Given the limited sample size, the very homogenous group of adults, and the imbalance in sex ratio, the effects of both sex, age, and BMI were not included in our data analysis. Therefore, our findings are explorative and should be validated in larger cohorts.

Previous studies have reported that the gut microbiome contributes to explaining PPGR to identical foods (1, 3–5). The recent PREDICT study, including more than 1,000 individuals, found that the gut microbiome composition, derived from 16S rRNA high-throughput sequencing of baseline stool samples, explained 6% of postprandial glycemia and that meal composition, genetics, meal context (i.e., meal timing, exercise, sleep, and circadian rhythm), and serum glycemic markers are more important determinants of postprandial glycemia (5). In light of our limited sample size, this explains why we were not able to identify any specific taxa of the gut microbiome being significantly associated with variability in PPGR in healthy subjects. Given that glucose mainly is absorbed in the small intestine, the influence of the colonic microbiome on glucose homeostasis is most likely to occur indirectly through production of SCFA and bile acid metabolism (25). However, even elimination of the gut microbiota with antibiotics has little or no effect on glucose metabolism in humans (26, 27), suggesting that the influence of the colonic microbiome on PPGR may be subtle. Altogether, this suggests that the gut microbiome mainly contributes to predictive models of PPGR because the gut microbiome reflects the individual's long-term dietary practices (28, 29), physical activity (30), intake of medicine (31), and differences in ITT (9).

Another modifiable variable, which could possibly influence glucose homeostasis, is ITT (32). Today, there is little consensus on the gold standard for measuring ITT. Scintigraphy (33), wireless SmartPills (34), and radio-opaque marker (9) methods are commonly used to determine ITT. However, other less expensive alternatives include a tasteless, non-absorbable, blue dye (35) and stool consistency assessed by BSS, a validated surrogate measure of ITT (15). Here, we found that ITT, estimated by sweet corn transit time, is commensurate with literature values obtained from wireless motility capsules (36) and correlated well with both stool consistency and fecal water content, confirming that this is a cheap and suitable alternative for estimating ITT. Also, fecal water content was as expected strongly correlated with BSS, suggesting that determination of fecal water may be used as a continuous and more objective measure of ITT rather than BSS, which is a subjective measure of stool consistency.

We observed a negative association between ITT and fasting glucose. While small intestinal motility and flow of luminal content are determinants of glucose absorption (37), little is known about the relation between ITT and glucose homeostasis (32). Numerous appetite hormones secreted from enteroendocrine cells in the intestine such as cholecystokinin (CCK), glucagon-like peptide 1 (GLP-1), and peptide YY (PYY), can regulate intestinal motility, satiety, and gastric emptying and thereby regulate glucose homeostasis (25). Therefore, such hormones could be considered in future mechanistic studies aimed at establishing the role of ITT in determining glucose homeostasis.

ITT and stool consistency have previously been associated with gut microbial composition, diversity, and metabolism (9, 11, 38). Here, we found ITT and a firm stool to be positively associated with the relative abundance of *Coprococcus* and fecal concentrations of branched SCFAs and furthermore a firm stool to be positively associated with fecal wet energy density and the relative abundance of *Blautia* and negatively associated with *Lachnospira*. This is in agreement with studies showing that amino acid fermentation increases in the distal colon when carbohydrate is depleted following a prolonged transit time (9, 39). Of notice, strong correlations were observed between *Coprococcus, Dialister*, and branched SCFAs. Previous studies have reported that these species produce SCFAs (40, 41). However, it remains unknown whether these species also produce branched SCFAs.

Finally, we observed that increased fasting insulin levels were associated with increased fasting breath hydrogen levels. Breath hydrogen exhalation reflects colonic fermentation (42) and studies have shown that ingestion of complex carbohydrates increases breath hydrogen exhalation (43, 44). In the current study, participants consumed a standardized evening meal containing lentils before fasting, prior to the test day, which was confirmed by the presence of tryptophan betaine in the blood. As lentils are high in resistant starch and fermented by the colonic microbiota (45), breath hydrogen measured on the test day likely, at least partly, reflected colonic fermentation of the lentils. Therefore, we speculate that individuals with increased colonic fermentation, as reflected by breath hydrogen, could have an increased release and absorption of glucose into the blood, resulting in more insulin being released into the bloodstream in the fasting state. In contrast, concentrations of fecal SCFAs were most likely not related to the evening meal before, since the stool sample was collected prior or shortly after the evening meal or in the early morning on the test day. This may explain why fecal SCFAs were not associated with fasting insulin. The inconsistent correlations between fasting insulin and these two different measures of colonic fermentation suggest that fecal SCFAs and breath hydrogen are not necessarily reflecting colonic fermentation at the same point in time.

In conclusion, we did not observe any associations between PPGRs and the gut microbial diversity nor with any specific taxa of the gut microbiome, measures of colonic fermentation, or abiotic factors in healthy adults, suggesting that the contributions of the gut microbiome and abiotic factors to PPGR may be subtle in healthy adults. We did however observe associations between ITT and fasting glucose, as well as between fasting breath hydrogen and fasting insulin, which could be used to generate new hypotheses for mechanistic research on the complex interactions between ITT, gut microbiome, and glucose homeostasis.

## Data Availability Statement

The datasets presented in this study can be found in online repositories. The names of the repository/repositories and accession number(s) can be found below: NCBI, PRJNA665627 (https://www.ncbi.nlm.nih.gov/search/all/?term=PRJNA665627).

## Ethics Statement

The studies involving human participants were reviewed and approved by the Ethical Committee of the Capital Region of Denmark. The patients/participants provided their written informed consent to participate in this study.

## Author Contributions

LD and HR: conception and design of the study. NN and JH: recruitment, collection of data, and biological samples. NN, LH, LK, and DN: fecal microbiota. JH and MB: fecal energy, fecal pH, fecal water determination. HR: liquid chromatography mass spectrometry. JH: fecal SCFAs. NN, JH, and HR: data analysis and interpretation and manuscript drafting. All authors read, revised and approved the final manuscript.

## Conflict of Interest

The authors declare that the research was conducted in the absence of any commercial or financial relationships that could be construed as a potential conflict of interest.
