# Supplementary material for: The Gut Microbiome and Abiotic Factors as Potential Determinants of Postprandial Glucose Responses: A Single-Arm Meal Study
Source: Front Nutr. 2021 Jan 14;7:594850. doi: 10.3389/fnut.2020.594850 (PMC7874175; doi:10.3389/fnut.2020.594850)
Supplement: Supplementary file 1 [file Data_Sheet_1.pdf]

Supplementary Material

1 Supplementary Tables

Supplementary Table 1. Energy and macronutrient composition of the standardised test meal

Food	Amount (g)	Energy (kJ)	Carbohydrate (g)	Protein (g)	Fat (g)
Orange juice	250	457.5	22.5	1.8	1.3
White bread	60	646.2	28.7	4.7	1.7
Butter	8	223.3	0.05	0.04	6.0
Raspberry jam	20	172	9.6	0.1	0.1
**Total *Standardised* Meal[1]**	**338**	**1499**	**60.9**	**6.6**	**9.1**

kJ = kilo Joule; g = gram; [1] Calculated with Vitakost.dk

2 Supplementary Figures

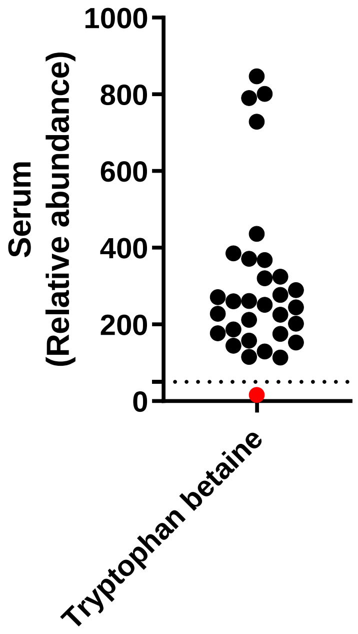

Supplementary Figure 1. Compliance with standardised evening meal containing lentils. Relative abundance of tryptophan betaine in serum of all 31 subjects. The metabolite was detected in considerable amounts in all subjects except one, which was assumed to be non-compliant (marked red). The relative abundance of tryptophan betaine was measured in all six serum samples obtained from each subject and the mean of these values are presented in the figure.

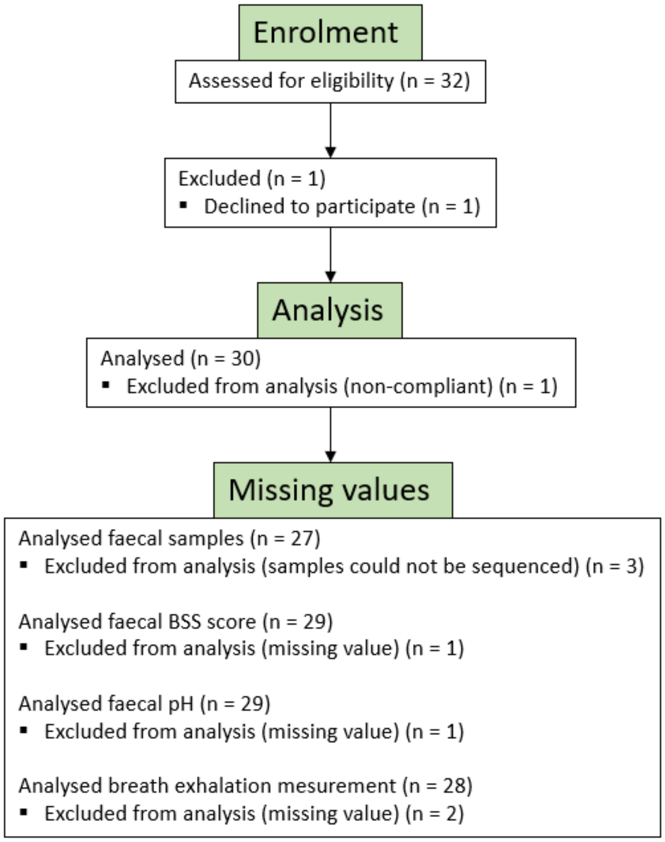

Supplementary Figure 2. Flow diagram. The flow of the participants from enrolment until analysis is shown. Number of participants (n).

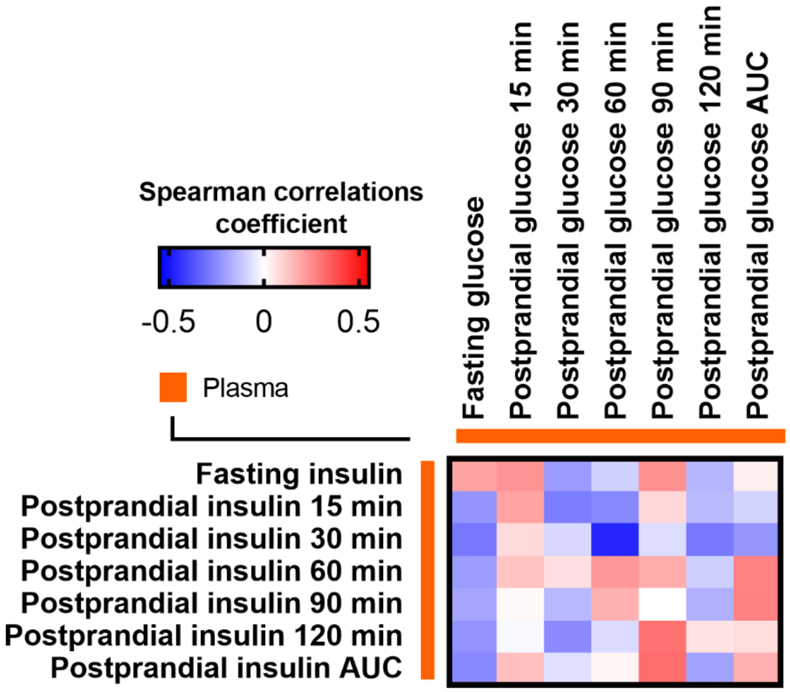

Supplementary Figure 3. Weak correlations between fasting and postprandial glucose and insulin measures upon the standardized breakfast across all individuals. No correlations remained significant after correction for multiple testing. Correlations were calculated based on Spearman's rank correlation. (n=30)

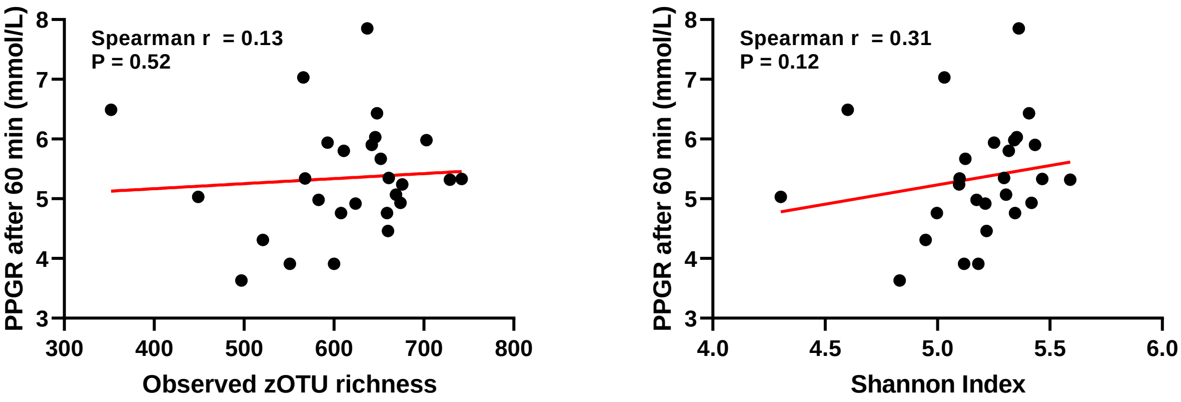

Supplementary Figure 4. Correlations between α-diversity and the postprandial glucose responses (PPGR) 60 minutes after the meal. Associations between PPGR 60 minutes after the meal and faecal bacterial α-diversity assessed by **(A)** observed zOTU richness and **(B)** and Shannon index, respectively. Correlations were calculated based on Spearman's rank correlation (n = 27).

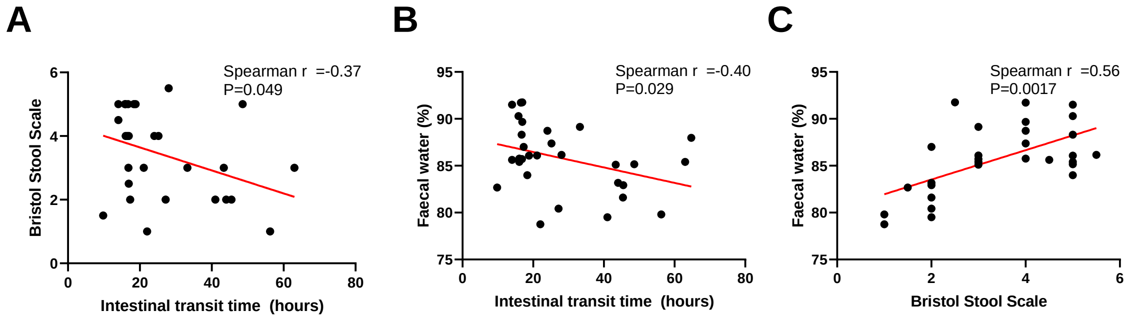

Supplementary Figure 5. Associations between measures of intestinal transit time. Associations between sweet corn's intestinal transit time and **(A)** stool consistency assessed by the Bristol Stool Scale and **(B)** faecal water, respectively. **(C)** Associations between stool consistency assessed by the Bristol Stool Scale and faecal water.

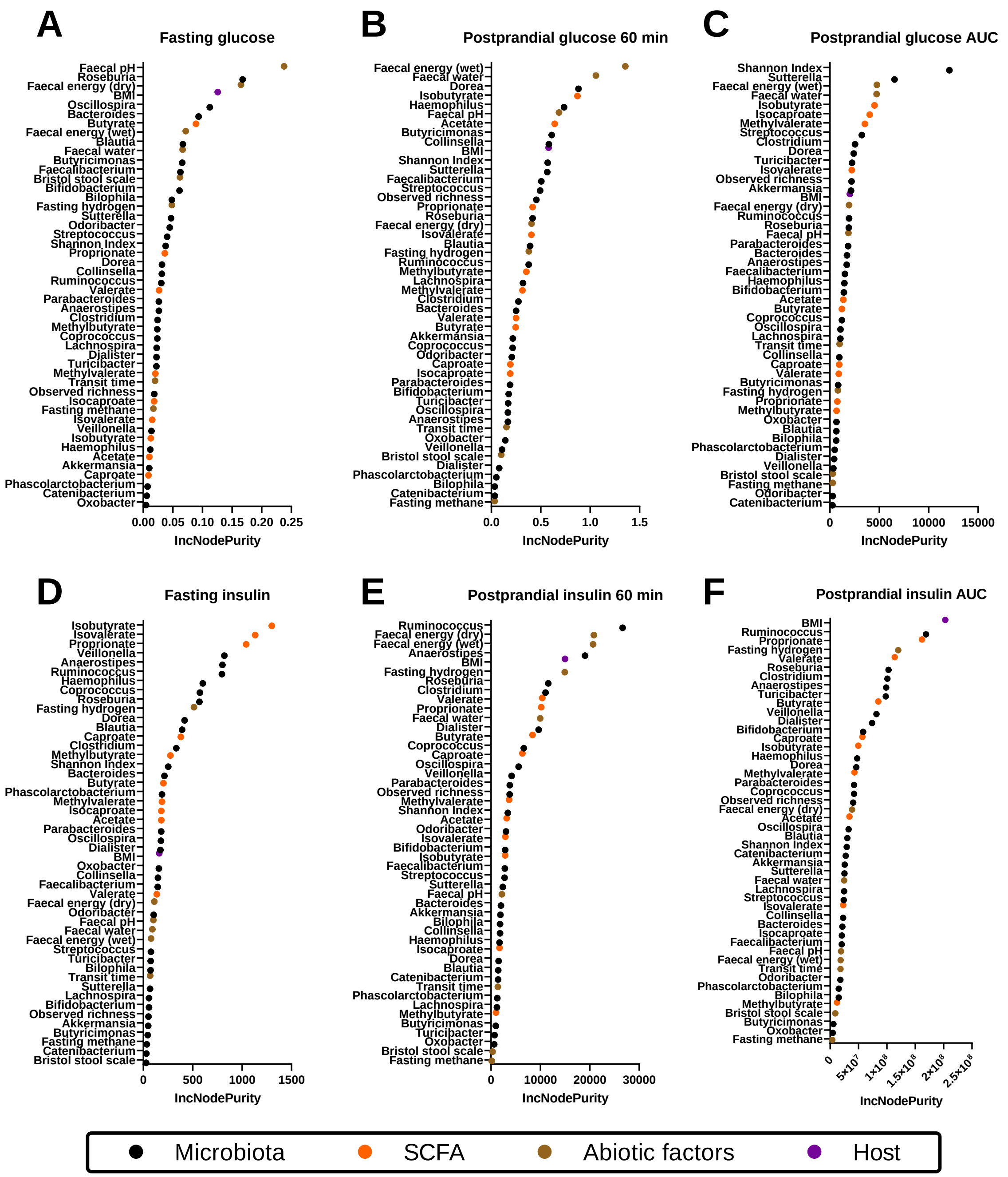

Supplementary Figure 6. Random Forest estimation of variable importance for predicting fasting and postprandial glucose and insulin responses. Mean Decrease Gini (IncNodePurity) sorted decreasingly from top to bottom of host, microbiota, short-chain fatty acids (SCFA) and abiotic variables as assigned by the random forest for (**A**) fasting glucose, (**B**) postprandial glucose at 60 min, (**C**) postprandial glucose AUC, (**D**) fasting insulin, (**E**) postprandial insulin at 60 min, and (**F**) postprandial insulin AUC, respectively. All individuals with complete data were included (n=26).
